# TNF-α-Induced cPLA_2_ Expression via NADPH Oxidase/Reactive Oxygen Species-Dependent NF-κB Cascade on Human Pulmonary Alveolar Epithelial Cells

**DOI:** 10.3389/fphar.2016.00447

**Published:** 2016-11-25

**Authors:** Chih-Chung Lin, Wei-Ning Lin, Rou-Ling Cho, Chen-yu Wang, Li-Der Hsiao, Chuen-Mao Yang

**Affiliations:** ^1^Department of Anesthetics, Chang Gung Memorial Hospital at Linkou and College of Medicine, Chang Gung UniversityTao-Yuan, Taiwan; ^2^Graduate Institute of Basic Medicine, Fu Jen Catholic UniversityNew Taipei City, Taiwan; ^3^Department of Physiology and Pharmacology and Health Aging Research Center, College of Medicine, Chang Gung UniversityTao-Yuan, Taiwan; ^4^Research Center for Industry of Human Ecology, Research Center for Chinese Herbal Medicine, and Graduate Institute of Health Industry Technology, Chang Gung University of Science and TechnologyTao-Yuan, Taiwan

**Keywords:** ASK1, cytokines, cytosolic phospholipase A_2_, lung inflammation, signaling transduction

## Abstract

Tumor necrosis factor-α (TNF-α) triggers activation of cytosolic phospholipase A_2_ (cPLA_2_) and then enhancing the synthesis of prostaglandin (PG) in inflammatory diseases. However, the detailed mechanisms of TNF-α induced cPLA_2_ expression were not fully defined in human pulmonary alveolar epithelial cells (HPAEpiCs). We found that TNF-α-stimulated increases in cPLA_2_ mRNA (5.2 folds) and protein (3.9 folds) expression, promoter activity (4.3 folds), and PGE_2_ secretion (4.7 folds) in HPAEpiCs, determined by Western blot, real-time PCR, promoter activity assay and PGE_2_ ELISA kit. These TNF-α-mediated responses were abrogated by the inhibitors of NADPH oxidase [apocynin (APO) and diphenyleneiodonium chloride (DPI)], ROS [N-acetyl cysteine, (NAC)], NF-κB (Bay11-7082) and transfection with siRNA of ASK1, p47*^phox^*, TRAF2, NIK, IKKα, IKKβ, or p65. TNF-α markedly stimulated NADPH oxidase activation and ROS including superoxide and hydrogen peroxide production which were inhibited by pretreatment with a TNFR1 neutralizing antibody, APO, DPI or transfection with siRNA of TRAF2, ASK1, or p47*^phox^*. In addition, TNF-α also stimulated p47*^phox^* phosphorylation and translocation in a time-dependent manner. On the other hand, TNF-α induced TNFR1, TRAF2, ASK1, and p47*^phox^* complex formation in HPAEpiCs, which were attenuated by a TNF-α neutralizing antibody. We found that pretreatment with NAC, DPI, or APO also attenuated the TNF-α-stimulated IKKα/β and NF-κB p65 phosphorylation, NF-κB (p65) translocation, and NF-κB promoter activity in HPAEpiCs. Finally, we observed that TNF-α-stimulated NADPH oxidase activation and ROS generation activates NF-κB through the NIK/IKKα/β pathway. Taken together, our results demonstrated that in HPAEpiCs, up-regulation of cPLA_2_ by TNF-α is, at least in part, mediated through the cooperation of TNFR1, TRAF2, ASK1, and NADPH oxidase leading to ROS generation and ultimately activates NF-κB pathway.

## Introduction

The occurrence and exacerbation of lung diseases, including chronic obstructive pulmonary disease (COPD) and asthma, is dependent on the severity of lung inflammation (Lee and Yang, 2012). Eicosanoids, one of lipid mediators generating from conversion of arachidonic acid (AA), have been found *in situ* in airway secretion of asthmatics (Barnes, 1989; Henderson et al., 2002). Phospholipase A_2_ (PLA_2_) enzymes catalyze the hydrolysis of membrane phospholipids resulting in the release of AA (Borsch-Haubold et al., 1998). The constitutive enzyme cyclooxygenase (COX)-1 or the inducible COX-2 then converts AA to prostaglandins (PGs), such as PGE_2_ (Yang et al., 2002; Hsieh et al., 2006). Three PLA_2_ have been identified including secretory PLA_2_, the 85 kDa cytosolic group IV PLA_2_ (cPLA_2_), and a calcium-independent group VI PLA_2_ in mammalian cells (Six and Dennis, 2000). cPLA_2_ plays a major role in agonist-induced AA release and eicosanoid production (Leslie, 1997). Involvement of cPLA_2_ in sepsis-related acute lung injury (Nagase et al., 2000) and anaphylaxis-associated bronchial reactivity has been proved (Uozumi et al., 1997). Furthermore, PGE_2_ synthesis increases are dependent on upregulation of cPLA_2_ activity in various cell types (Dieter et al., 2002; Gilroy et al., 2004). Elevated levels of TNF-α have been detected in the bronchoalveolar lavage fluid of asthmatic patients. TNF-α could exaggerate inflammatory responses through up-regulation of inflammatory genes, such as cPLA_2_ (Hulkower et al., 1994; Van Putten et al., 2001). Up-regulation of cPLA_2_ further catalyzes the hydrolysis of membrane phospholipids and releases AA served as a substrate for PGs synthesis (i.e., PGE_2_) that augments lung inflammation. Moreover, our previous findings also provided insights into the correlation between COX-2 and cPLA_2_ expression in ATPγS-stimulated vascular smooth muscle cells (VSMCs) with similar molecular mechanisms and functional coupling to amplify the occurrence of vascular inflammation (Lin et al., 2009). Therefore, the synthesis of PGE_2_ could be a good index of AA release that is more sensitive than [^3^H]AA mobilization (Berenbaum et al., 2003). In this study, although the effect of TNF-α on COX-2 expression was not investigated, we tested the effect of TNF-α on PGE_2_ synthesis as a parameter of cPLA_2_ activity in human pulmonary alveolar epithelial cells (HPAEpiCs). Therefore, up-regulation of cPLA_2_ may play a key role in local and systemic inflammation in airway diseases. However, the molecular mechanisms by which TNF-α induces cPLA_2_ expression and PGE_2_ synthesis in HPAEpiCs are not completely understood.

Previous report indicates that TNF-α binds to distinct receptors, TNFR1 and TNFR2, and triggers various inflammatory responses (Lee et al., 2009). The association of TNF-α and TNFR1 modulates the severity of tissue injury via activation of proinflammatory or programmed cell death pathway (van Vliet et al., 2005; Lee et al., 2009). TNF receptor associated factor 2 (TRAF2) plays an important role in innate immune and inflammatory responses. However, the interaction among TNF-α, TNFR1, TRAF2 and downstream components leading to cPLA_2_ expression is still unknown in HPAEpiCs.

Reactive oxygen species (ROS) are products of normal cellular metabolism acting as second messengers (Lee and Yang, 2012). However, either reduced nicotinamide adenine dinucleotide phosphate (NADPH) by pro-inflammatory cytokines such as TNF-α or the mitochondrial electron transport chain and xanthine oxidase leads to increased production of ROS and unbalance of cellular oxidative stress, which are causes of airway/lung damages and subsequently respiratory inflammatory diseases/injuries (Lee and Yang, 2012). Apoptosis signal-regulating kinase 1 (ASK1), a mitogen-activated protein kinase kinase kinase, participates in regulating stress and immune responses. ASK1 is activated by cytokines and various environmental and cellular stresses. Hsu et al. indicated that peptidoglycan (PGN) induced COX-2 expression via an ASK1 signaling in A549 cells (Hsu et al., 2010). Therefore, we explored whether TNFR1, TRAF2, ASK1, and NADPH oxidase/ROS are involved in TNF-α-induced cPLA_2_ expression and PGE_2_ release.

NF-κB plays major roles not only in the evolution but also in the resolution of inflammatory responses. A wide spectrum of biological effects including immune and stress-induced responses, proliferation, differentiation, tumorigenesis, apoptosis, and tissue remodeling are all controlled by activated NF-κB (Lee and Yang, 2012). The activation of NF-κB can be regulated by various extracellular stimuli, including cytokines and oxidative stress (Lee and Yang, 2012). We noticed that ROS generation can impact NF-κB signaling pathways (Morgan and Liu, 2011). In addition, NF-κB modulates cPLA_2_ gene activity in various cell types (Luo et al., 2006; Huwiler et al., 2012; Chi et al., 2014). Therefore, we examined whether TNF-α regulates cPLA_2_ expression via ROS-dependent NF-κB activation in HPAEpiCs.

In addressing these questions, the experiments were performed to investigate the mechanisms underlying TNF-α-induced cPLA_2_ expression and PGE_2_ synthesis in HPAEpiCs. These findings suggested that TNF-α-induced cPLA_2_-expression associated PGE_2_ release is, at least in part, mediated through a TNFR1/TRAF2/ASK1/p47*^phox^*/NADPH oxidase/ROS/NIK/IKKα/β/NF-κB pathway in these cells.

## Materials and Methods

### Materials

Recombinant human TNF-α was from R&D System (Minneapolis, MN, USA). Anti-cPLA_2_ (sc-454), anti-p47*^phox^* (sc-14015), anti-Gαs (sc-823), anti-TRAF2 (sc-7346), anti-ASK1 (sc-5294), anti-TNFR1 (sc-52739), anti-NIK (sc7211), anti-IKKα (sc7218), anti-IKKβ (sc8014), anti-p65 (sc-7151) and anti-phospho-serine (sc-81515) antibodies were from Santa Cruz (Santa Cruz, CA). An anti-GAPDH antibody (#MCA-1D4) was from Encor (Gainesville, FL, USA). Human TNF-α neutralizing antibody (#7321), anti-phospho-tyrosine (#9411), anti-phospho-ASK1 (#3765), anti-phospho-p65 (#3031), and anti-phospho-IKKα/β (#2697) antibodies were from Cell Signaling (Danvers, MA, USA). *N*-Acetyl cysteine (NAC), diphenyleneiodonium chloride (DPI), apocynin (APO), and Bay11-7082 were from Biomol (Plymouth Meeting, PA, USA). Dihydroethidium (DHE) and 5-(and-6)-chloromethyl-2′,7′-dichlorodihydrofluorescein diacetate, acetyl ester (CM-H_2_DCFDA) were from Molecular Probes (Eugene, OR, USA). SDS-PAGE supplies were from MDBio Inc (Taipei, Taiwan). All other reagents were from Sigma (St. Louis, MO, USA).

### Cell Culture and Treatment

Human pulmonary alveolar epithelial cells (HPAEpiCs) were ordered from ScienCell Research Lab (San Diego, CA, USA). The passages 4–7 were used throughout this study. HPAEpiCs were cultured in DMEM/F12 medium containing 10% FBS, as previously described Lee et al. (2008). The growth medium was changed after 48 h and then every 3 days. The viability of HPAEpiCs after treatment with DMSO or the pharmacological inhibitors alone was determined by an XTT [2,3-bis-(2-methoxy-4-nitro-5-sulfophenyl)-2H-tetrazolium-5-carboxanilide] assay, which showed no significant differences (data not shown).

### Western Blot Analysis

Serum-starved HPAEpiCs were incubated with TNF-α at 37°C for the various time points. At the end of treatment, the cells were harvested and centrifuged at 45000 × *g* at 4°C for 1 h to prepare the whole cell lysate, as previously described (Chi et al., 2012). The denatured samples were analyzed by 10% SDS-PAGE gels and transferred to nitrocellulose membrane. The Western blot was performed by incubation membrane with an anti-cPLA_2_, anti-p47*^phox^*, anti-Gαs, anti-TRAF2, anti-ASK1, anti-TNFR1, anti-NIK, anti-IKKα, anti-IKKβ, anti-p65, anti-phospho-serine or anti-GAPDH (1:1000) antibody for 24 h, and then incubated with an anti-mouse horseradish peroxidase antibody (1:2000) for 1 h. ECL reagents and UVP BioSpectrum 500 Imaging System (Upland, CA, USA) were used to detect and capture the immunoreactive bands. The image densitometry of each immunoreactive band was analyzed and quantified by the UN-SCAN-IT gel software (Orem, UT, USA).

### Real-Time PCR Analysis

Total RNA of HPAEpiCs was extracted using TRIzol reagent and reverse-transcribed into cDNA. Real-time PCR using SYBR Green PCR reagents (Applied Biosystems, Branchburg, NJ, USA) and primers specific for cPLA_2_ and GAPDH genes were used, as previously described (Chi et al., 2012). The real-time primers were as follows: cPLA_2_α, forward primer: 5′-ATGATAGCTCGGACAGTGATGATGA-3′; reverse primer: 5′-CATACGATGAATCCAACTTGCTTGA-3′ and GAPDH, forward primer: 5′-CTCTGCTCCTCCTGTTCGAC-3′; reverse primer: 5′-TTAAAAGCAGCCCTGGTGAC-3′. The expression of cPLA_2_ was quantified by normalization to the GAPDH expression.

### Measurement of Intracellular ROS Accumulation

The intracellular H_2_O_2_ levels were determined by measuring fluorescence of 2′,7′-dichloro fluorescein diacetate (DCF-DA) and the $O_{2}^{•–}$ levels were determined by measuring the level of dihydroethidium (DHE), as previously described Hsu et al. (2014). The fluorescence for DCF and DHE staining was detected at 495/529 and 518/605 nm, respectively, using a fluorescence microscope (Zeiss, Axiovert 200M). For the purpose of these experiments, HPAEpiCs were washed with warm HBSS and incubated in HBSS or cell medium containing 10 μM DCFH-DA or DHE at 37°C for 45 min. Subsequently, HBSS or medium containing DCFH-DA or DHE was removed and replaced with fresh medium. HPAEpiCs were then incubated with TNF-α. Cells were washed twice with PBS and detached with trypsin/EDTA, and the fluorescence intensity of the cells was analyzed using a multi-technology reader (Thermo, Appliskan) at Ex/Em: 485/530 nm.

### Determination of NADPH Oxidase Activity by Chemiluminescence Assay

HPAEpiCs (2 × 10^6^ cells/ml) were cultured in 6-well plates and then incubated with TNF-α after growth to confluence and serum-starved. At the end of incubation, cells were harvested and centrifuged at 400 × *g* for 10 min at 4°C. The cell pellet was kept on ice after re-suspended by 35 μl/per well of ice-cold RPMI-1640 medium. The complex including NADPH (1 μM) or lucigenin (20 μM), 5 μl of cell suspension (0.2 × 10^5^ cells) and a final 200 μl volume of pre-warmed (37°C) RPMI-1640 medium were prepared and the chemiluminescence was immediate recorded with an Appliskan luminometer (Thermo^®^) in out-of-coincidence mode. Appropriate blanks and controls were used. There was no background chemiluminescence of lucigenin in NADPH or NADH alone group (30–40 counts per min), as previously described Hsu et al. (2014). The signals of chemiluminescence were continuously measured for 12 min, and the activity of NADPH oxidase was expressed as counts per million cells.

### Measurement of cPLA_2_ Luciferase Activity

Human cPLA_2_ promoter spanning - 2375 to +75 bp as cloned into pGL3-basic vector (Promega, Madison, WI, USA) as cPLA_2_-luc plasmid. The activity of cPLA_2_-luc was detected using a luciferase assay system (Promega, Madison, WI, USA), as previously described Chi et al. (2012). The detected luciferase activities were standardized with the activity of β-gal.

### Measurement of PGE_2_ Generation

The serum-starved cells were treated with TNF-α for the different time points. At the end of treatment, culture media were collected and stored at -80°C. The concentrations of PGE_2_ were detected by a PGE_2_ enzyme immunoassay kit (Cayman) according to the manufacturer’s instructions, as previously described Lee et al. (2008).

### Transient Transfection with siRNAs

All human ASK1, p47*^phox^*, TRAF2, NIK, IKKα, IKKβ, and p65 siRNA together with scramble siRNA were purchased from Sigma (St. Louis, MO, USA). Lipofectamine^TM^ RNAiMAX reagents were used to prepare siRNA liposome complexes (100 nM of siRNAs) according to the manufacturer’s instructions, as previously described Lee et al. (2008).

### Co-immunoprecipitation Assay

Cell lysates containing 1 mg of protein were incubated with 2 μg of an anti-p47*^phox^*, anti-TNFR1, anti-TRAF2, or anti-ASK1 antibody at 4°C for 24 h, and then 10 μl of 50% protein A-agarose beads was added and mixed at 4°C for 24 h. The immunoprecipitates were collected and washed thrice with a lysis buffer without Triton X-100. 5x Laemmli buffer was added and subjected to electrophoresis on SDS-PAGE, and then blotted using an anti-phospho-tyrosine, anti-phospho-serine, anti-p47*^phox^*, anti-TRAF2, anti-TNFR1, or anti-ASK1 antibody, as previously described (Yang et al., 2014).

### Immunofluorescence Staining

Growth-arrested HPAEpiCs were incubated with TNF-α for the indicated time intervals. After washing twice with ice-cold PBS, cells were fixed, permeabilized and stained using an anti-p65 antibody, as previously described (Chi et al., 2011). A fluorescence microscope (Zeiss, Axiovert 200M) were used to observe images.

### Cell Fractions Isolation

The cell lysates were sonicated for 5 s at output 1.5 using a sonicator (Misonix, Farmingdale, NY, USA) and then centrifuged at 6800 × *g* for 15 min at 4°C, as previously described Lee et al. (2008). The pellet was collected as the nuclear fraction. The supernatant was further centrifuged at 20,000 × *g* at 4°C for 60 min to yield the pellet (membrane fraction) and the supernatant (cytosolic fraction).

### Statistical Analysis of Data

Data were showed as the mean or mean ± SEM of five individual experiments and estimated using GraphPad Prism Program (GraphPad, San Diego, CA, USA). All the data were analyzed by paired two-tailed Student’s *t*-test or by one-way analysis of variance (ANOVA) followed with Tukey’s *post hoc* test at *p* < 0.05 level of significance.

## Results

### TNF-α Induces cPLA_2_ Expression via NADPH Oxidase and ROS

**FIGURE 1:**
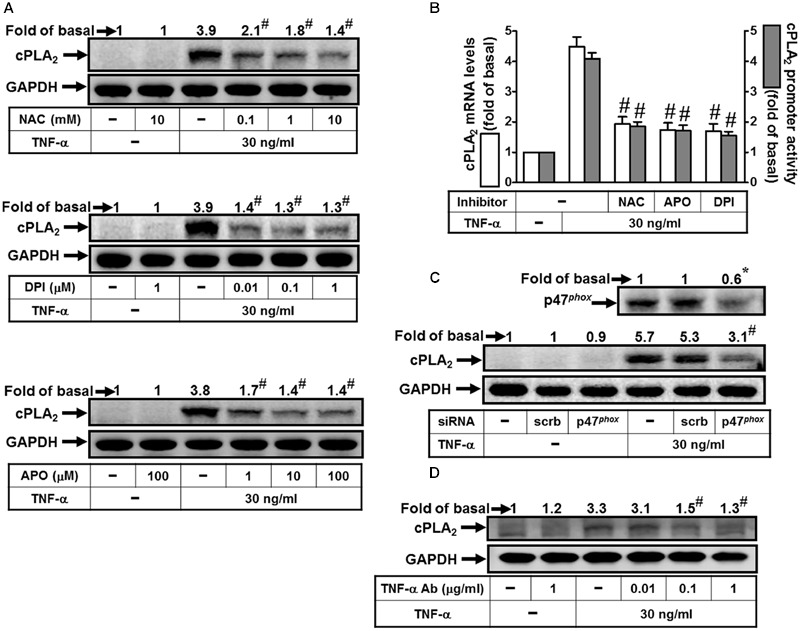
**TNF-α induces NADPH oxidase- and ROS-dependent cPLA_2_ expression. (A)** HPAEpiCs were pretreated with NAC, DPI, or APO for 1 h, and then incubated with TNF-α for 24 h. **(B)** Cells were pretreated with NAC (10 mM), DPI (1 μM), or APO (100 μM) for 1 h, and then incubated with TNF-α for 6 h. cPLA_2_ mRNA levels and promoter activity were determined. **(C)** Cells were transfected with scrambled or p47*^phox^* siRNA, and then incubated with TNF-α for 24 h. **(D)** HPAEpiCs were pretreated with human TNF-α neutralizing antibody (TNF-α nAb: 0.01, 0.1 and 1 μg/ml) for 1 h, and then incubated with TNF-α for 24 h. **(A,C,D)** The protein levels of cPLA_2_ and p47^phox^ were determined by Western blot. Data are expressed as mean ± SEM of three independent experiments. ^∗^*P* < 0.05, ^#^*P* < 0.01 as compared with the cells exposed to TNF-α alone.

Reactive oxygen species play both deleterious and beneficial roles. Accumulation of ROS and cellular oxidative stress trigger expression of inflammatory genes and result in tissue damages and various diseases (Lee and Yang, 2012). Thus, we attempted to investigate the roles of NADPH Oxidase and ROS in cPLA_2_ expression. Here we reported that TNF-α-induced cPLA_2_ protein levels were significantly reduced by pretreatment with a ROS scavenger (NAC) or the inhibitors of NADPH oxidase (APO and DPI) (**Figure 1A**). In addition, pretreatment with NAC, DPI, or APO also attenuated the TNF-α-stimulated cPLA_2_ mRNA expression and promoter activity (**Figure 1B**). The p47*^phox^* protein, one of cytosolic subunits of NADPH oxidase, contributed to acute NADPH oxidase activation via being phosphorylated and binding to p22*^phox^* (Lee et al., 2009). Thus, we confirmed the role of p47*^phox^* in cPLA_2_ expression by transfection with p47*^phox^* siRNA which knocked down protein expression of p47*^phox^* and then markedly inhibited TNF-α-induced cPLA_2_ protein expression in these cells (**Figure 1C**). To confirm this cPLA_2_ expression is mediated through TNF-α-dependent induction, HPAEpiCs were pretreated with a human TNF-α neutralizing antibody followed by TNF-α treatment. We found that TNF-α neutralizing antibody significantly blocked the cPLA_2_ induction in a dose-dependent manner (**Figure 1D**). These results indicated that TNF-α induces cPLA_2_ expression via NADPH oxidase and ROS in HPAEpiCs.

### TNF-α Induces NADPH Oxidase-Dependent Superoxide and Hydrogen Peroxide Production

**FIGURE 2:**
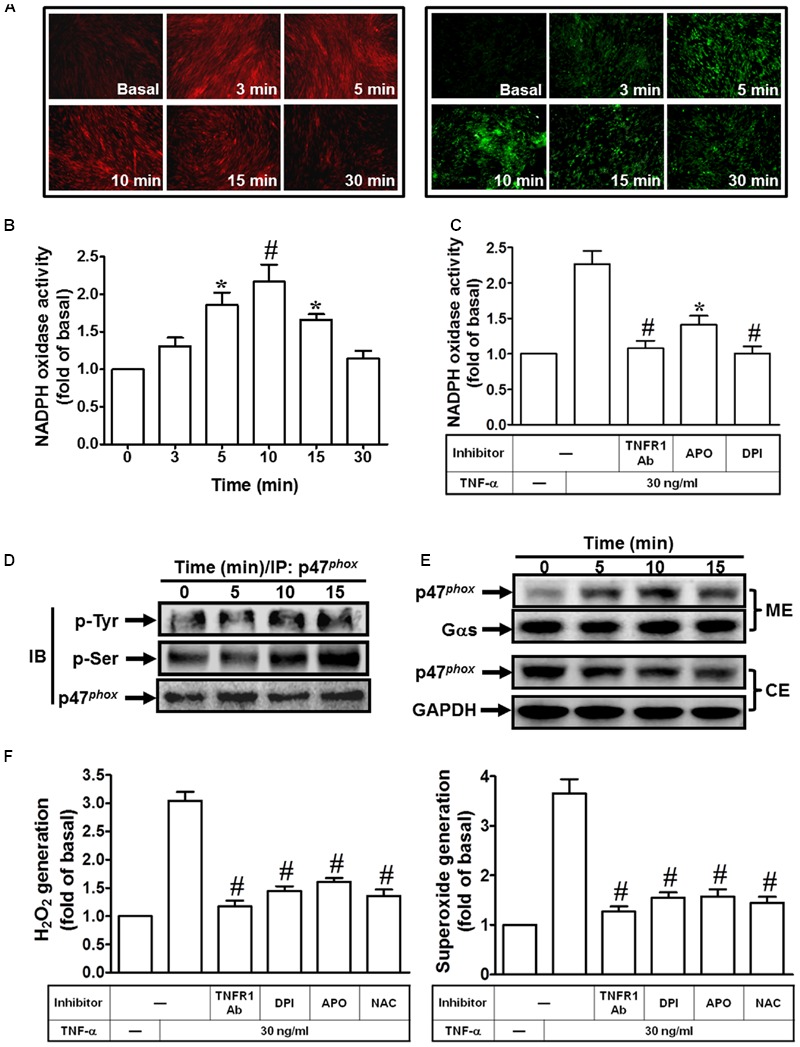
**TNF-α induces NADPH oxidase-dependent ROS generation. (A)** HPAEpiCs were treated with TNF-α for the indicated time intervals. DHE (red) or DCF (green) fluorescence image was observed. Images shown are representative of five independent experiments with similar results. Cells were **(B)** treated with TNF-α for the indicated time intervals or **(C)** pretreated with TNFR1 neutralizing antibody (10 μg/ml), APO (100 μM), or DPI (1 μM) for 1 h, and then treated with TNF-α for 10 min. NADPH oxidase activity was measured. **(D)** Cells were treated with TNF-α for the indicated time intervals. The cell lysates were subjected to immunoprecipitation using an anti-p47*^phox^* antibody, and then the immunoprecipitates were analyzed by Western blot using an anti-phospho-tyrosine, anti-phospho-serine, or anti-p47*^phox^* antibody. **(E)** Cells were treated with TNF-α for the indicated time intervals. The cytosolic and membrane fractions were prepared and analyzed by Western blot using an anti-p47*^phox^*, anti-G_αs_, or anti-GAPDH antibody. GAPDH and Gαs were used as a marker protein for cytosolic and membrane fractions, respectively. **(F)** Cells were pretreated with TNFR1 neutralizing antibody, NAC, APO, or DPI for 1 h, and then incubated with TNF-α for 10 min. H_2_O_2_ and superoxide generation were measured. Data are expressed as mean ± SEM of three independent experiments. *^∗^P* < 0.05, ^#^*P* < 0.01 as compared with the cells exposed to vesicle alone **(B)** or TNF-α alone **(C,F)**.

TNF-α may stimulate ROS production by several sources, such as mitochondria, but recent studies have strongly suggested that a major source of ROS is a phagocyte-type NADPH oxidase. Several reports also demonstrate that TNF-α triggers several signal transduction pathways to activate the NOX activity and enhances intracellular ROS generation leading to expression of inflammatory genes (Rahman et al., 1998; Hashimoto et al., 2001; Li et al., 2005; Lee et al., 2013; Yang et al., 2014). Therefore, we investigated whether TNF-α-induced cPLA_2_ expression is due to activation of NADPH oxidase and ROS generation. Here, we found that TNF-α markedly induced superoxide and hydrogen peroxide production, determined by using DHE or DCF under a fluorescence microscope (**Figure 2A**). On the other hand, we also observed that TNF-α time-dependently induces NADPH oxidase activation (**Figure 2B**), which was reduced by TNFR1 neutralizing antibody, APO, or DPI (**Figure 2C**). These results suggested that TNF-α induced ROS generation via NADPH oxidase activation. The p47*^phox^* is phosphorylated on several serine residues within the polybasic region of the protein, and these multiple phosphorylation events induce conformational changes that permit p47*^phox^* to interact with the cytoplasmic tail of p22*^phox^* and to initiate the formation of the active NADPH oxidase complex (Dang et al., 2006). Previous report also indicates that Src modulates tyrosine phosphorylation of p47*^phox^* in hyperoxia-induced activation of NADPH oxidase and generation of ROS in lung endothelial cells (Chowdhury et al., 2005). Thus, we investigated whether TNF-α stimulates the phosphorylation of tyrosine or serine on p47*^phox^*. As shown in **Figure 2D**, TNF-α increased tyrosine and serine phosphorylation of p47*^phox^* in a time-dependent manner. Moreover, p47*^phox^* was translocated from the cytosol to the membrane fractions in TNF-α-stimulated cells (**Figure 2E**). Finally, we investigated whether TNF-α-induced superoxide and hydrogen peroxide production are mediated through NADPH oxidase activation. Here, we observed that TNF-α-induced superoxide and hydrogen peroxide production were inhibited by TNFR1 neutralizing antibody, APO, DPI and NAC (**Figure 2F**). These results suggested that TNF-α induces ROS generation via p47*^phox^* translocation and NADPH oxidase activation in HPAEpiCs.

### TNF-α Induces TNFR1, TRAF2, ASK1, and p47*^phox^* Complex Formation

**FIGURE 3:**
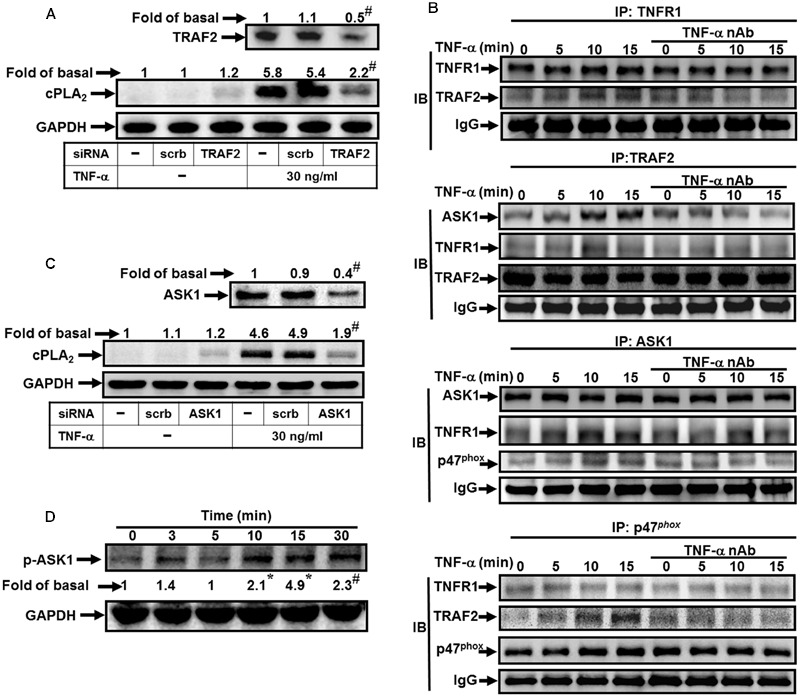
**TNF-α induces TNFR1/TRAF2/ASK1/p47*^phox^* complex formation. (A)** HPAEpiCs were transfected with scrambled or TRAF2 siRNA, and then incubated with TNF-α for 24 h. The protein levels of TRAF2 and cPLA_2_ were determined. **(B)** Cells were pretreated without or with human TNF-α neutralizing antibody (TNF-α nAb:1 μg/ml) for 1 h and then incubated with TNF-α for the indicated time intervals. The cell lysates were subjected to immunoprecipitation using an anti-TNFR1, anti-TRAF2, anti-ASK1, or anti-p47*^phox^* antibody, and then the immunoprecipitates were analyzed by Western blot by using an anti-TNFR1, anti-TRAF2, anti-ASK1, anti-p47*^phox^* or anti-IgG antibody. The results of IgG were used to be the loading control. **(C)** Cells were transfected with scrambled or ASK1 siRNA, and then incubated with TNF-α for 24 h. The protein levels of ASK1 and cPLA_2_ were determined. **(D)** Cells were treated with TNF-α for the indicated time intervals. The levels of phospho-ASK1 were determined by Western blot. Data are representative of three independent experiments with similar results. ^∗^*P* < 0.05, ^#^*P* < 0.01 as compared with the basal group.

Both tissue injury-related proinflammatory and programmed cell death pathways are activated by TNF-α via binding to TNFR1 (van Vliet et al., 2005; Lee et al., 2009). TRAF2 plays an important role in innate immune and inflammatory responses. Thus, we determine whether TRAF2 is a downstream component of TNF-α/TNFR1 complex in cPLA_2_ expression. Here, we demonstrated that TNF-α induced cPLA_2_ expression via TRAF2 by transfection with TRAF2 siRNA (**Figure 3A**). We found that transfection with TRAF2 siRNA reduced TRAF2 protein expression by about 50% and significantly attenuated the TNF-α-induced cPLA_2_ expression from 5.4-fold to 2.2-fold. In addition, we also showed that TNF-α stimulated TRAF2 and TNFR1 complex formation and its downstream components (**Figure 3B**). Therefore, we further studied whether TNF-α promoted the association of TNFR1, TRAF2, ASK1, and p47*^phox^* in HPAEpiCs. Data in **Figure 3B** showed that TNF-α time-dependently induced TNFR1, TRAF2, ASK1, and p47*^phox^* complex formation. Importantly, this interaction was blocked by pretreatment with TNF-α neutralizing antibody.

Various cell stresses, including ROS, TNF-α, lipopolysaccharide (LPS), and ER stress, activate ASK1 and resulting in apoptosis, differentiation and inflammation (Soga et al., 2012). However, whether ASK1 involved in TNF-α-mediated responses was still unknown in HPAEpiCs. Here, we found that transfection with ASK1 siRNA knocked down ASK1 protein level by about 50% and then significantly attenuated the TNF-α-induced cPLA_2_ expression from 4.9-fold to 1.9-fold (**Figure 3C**). ASK1 plays a pivotal role in ROS generation. In resting cells, endogenous ASK1 constitutively associated with thioredoxin (Trx) forming an inactive high-molecular-mass complex, known as ASK1 signalosome. Once cellular ROS increased, Trx dissociated from ASK1 signalosome and resulted in full activation of higher-molecular-mass complex by the recruitment of TRAF2 and TRAF6 (Fujino et al., 2007). Thus, we further investigated whether p47*^phox^* was recruited to the complex of TNF-α/TNFR1/TRAF2/ASK1. We observed that TNF-α time-dependently induced TNFR1, TRAF2, ASK1, and p47*^phox^* complex formation determined by immunoprecipitation (**Figure 3B**). Consistently, this interaction was also blocked by pretreatment with a TNF-α neutralizing antibody. To confirm whether ASK1 phosphorylation is necessary for TNF-α-induced cPLA_2_ expression, activation of the ASK1 was assayed by Western blot using an antibody specific for the phosphorylated form of ASK1. We found that TNF-α significantly stimulated ASK1 phosphorylation in a time-dependent manner (**Figure 3D**). These results suggested that TNF-α stimulates TNFR1, TRAF2, ASK1, and p47*^phox^* complex formation leading to NADPH oxidase activation and ROS generation in HPAEpiCs.

### TNF-α Induces NADPH Oxidase/ROS Generation and PGE_2_ Release via TRAF2, ASK1, and p47*^phox^*

**FIGURE 4:**
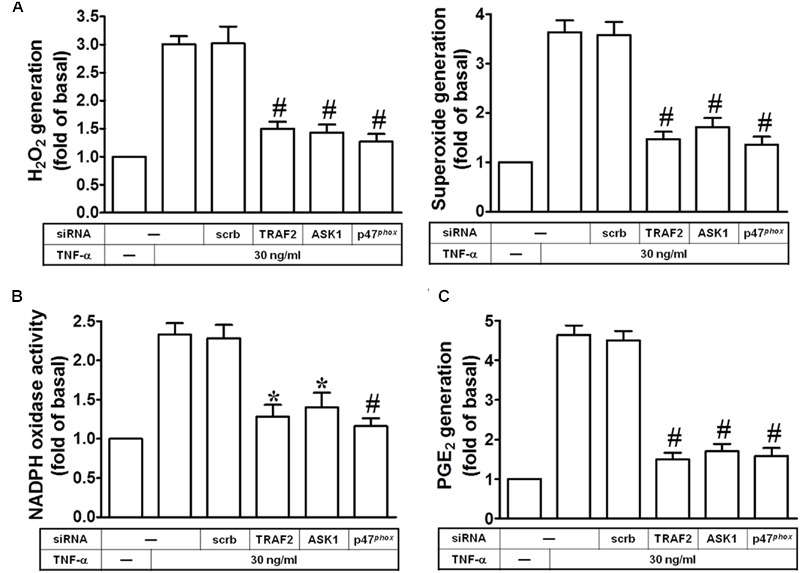
**TNF-α induces NADPH oxidase/ROS generation and PGE_2_ release via TRAF2, ASK1, and p47*^phox^*.** HPAEpiCs were transfected with scrambled, ASK1, p47*^phox^*, or TRAF2 siRNA, and then incubated with TNF-α for **(A,B)** 10 min or **(C)** 24 h. **(A)** H_2_O_2_ and superoxide generation were measured. **(B)** NADPH oxidase activity was measured. **(C)** PGE_2_ generation was measured. Data are expressed as mean ± SEM of three independent experiments. *^∗^P* < 0.05, ^#^*P* < 0.01 as compared with the cells exposed to TNF-α + scrambled siRNA.

Next, we investigated whether TRAF2, ASK1 and p47*^phox^* are involved in TNF-α-induced ROS generation. As shown in **Figure 4A**, transfection with TRAF2, ASK1, or p47*^phox^* siRNA markedly inhibited TNF-α-induced superoxide and hydrogen peroxide production. In addition, these siRNAs also inhibited the TNF-α-induced NADPH oxidase activation (**Figure 4B**). Moreover, our previous findings also provided the correlation between COX-2 and cPLA_2_ expression in ATPγS-stimulated VSMCs with similar molecular mechanisms and functional coupling to amplify the occurrence of vascular inflammation (Lin et al., 2009). Therefore, the synthesis of PGE_2_ could be a good index of AA release that is more sensitive than [^3^H]AA mobilization (Berenbaum et al., 2003). In this study, we tested the effect of TNF-α on PGE_2_ synthesis as a parameter of cPLA_2_ activity in HPAEpiCs. Here, we observed that TNF-α-induced PGE_2_ release was reduced by transfection with TRAF2, ASK1, or p47*^phox^* siRNA (**Figure 4C**). These results suggested that TNF-α induces ROS generation and PGE_2_ release via TRAF2, ASK1, and p47*^phox^* in HPAEpiCs.

### TNF-α Induces cPLA_2_ Expression via NIK, IKKα/β, and NF-κB

**FIGURE 5:**
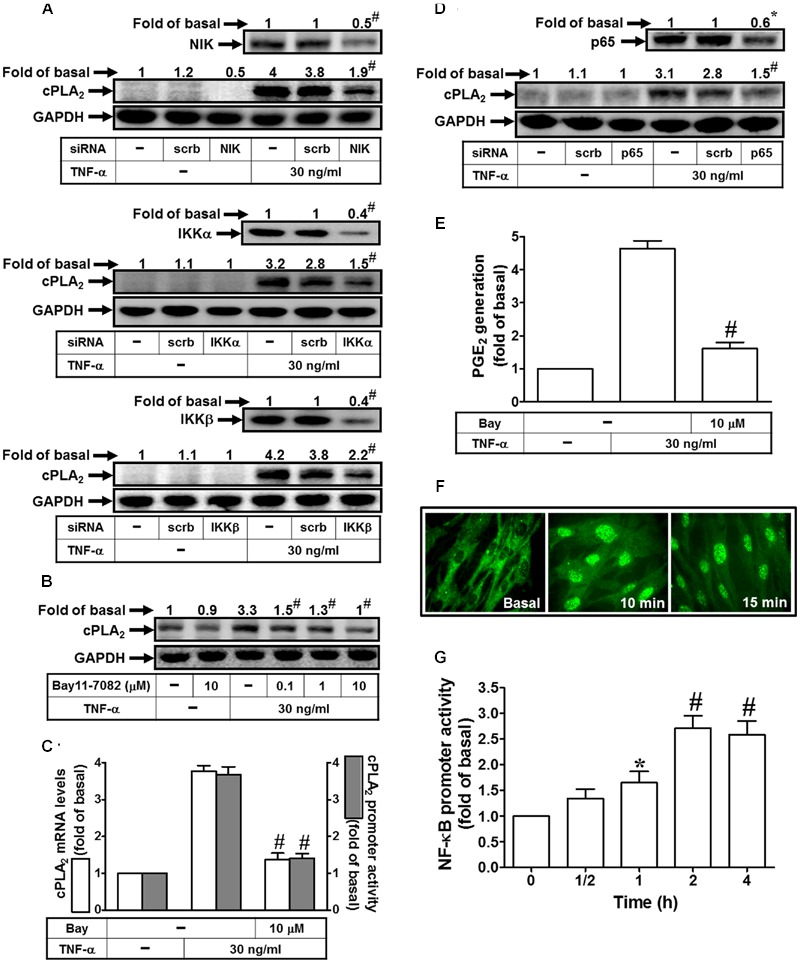
**TNF-α induces cPLA_2_ expression via NIK, IKKα/β, and NF-κB. (A)** HPAEpiCs were transfected with scrambled, NIK, IKKα, or IKKβ siRNA, and then incubated with TNF-α for 24 h. The protein levels of NIK, IKKα, IKKβ, and cPLA_2_ were determined by Western blot. **(B)** Cells were pretreated with Bay11-7082 for 1 h, and then incubated with TNF-α for 24 h. The protein levels of cPLA_2_ were determined by Western blot. **(C)** Cells were pretreated with Bay11-7082 for 1 h, and then incubated with TNF-α for 6 h. cPLA_2_ mRNA levels and promoter activity were determined. **(D)** Cells were transfected with scrambled or p65 siRNA, and then incubated with TNF-α for 24 h. The protein levels of p65 and cPLA_2_ were determined. **(E)** Cells were pretreated with Bay11-7082 for 1 h, and then incubated with TNF-α for 24 h. PGE_2_ generation was measured. **(F)** Cells were incubated with TNF-α for the indicated time intervals. Cells were fixed, labeled with an anti-p65 antibody, and then FITC-conjugated secondary antibody. Individual cells were imaged. **(G)** Cells were incubated with TNF-α for the indicated time intervals. NF-κB promoter activity was determined. Data are expressed as mean ± SEM of three independent experiments. *^∗^P* < 0.05, ^#^*P* < 0.01 as compared with the cells exposed to TNF-α alone **(C,E)** or vesicle alone **(G)**.

NF-κB is mainly involved in stress-induced immune and inflammatory responses. Activities of NF-κB are regulated by various inflammatory cytokines such as TNF-α (Lee et al., 2007). The signaling mechanisms mediated activation of NF-κB includes canonical and non-canonical pathways (Tak and Firestein, 2001). The canonical pathway has been well documented to regulate the pathophysiological functions; however, the non-canonical NF-κB-inducing kinase (NIK) pathway is not well understood in the expression of inflammatory genes (Uno et al., 2014). NIK plays central roles in the activation of non-canonical NF-κB pathway (Tak and Firestein, 2001; Uno et al., 2014). NIK was first identified as a TRAF2 interacting protein. In addition, IκBs associate with NF-κB being inactive forms. IκBs are phosphorylated by IKKα and IKKβ then degradation via ubiquitination to release NF-κB and nuclear translocation (Sun, 2011). Therefore, we investigated whether TNF-α induced cPLA_2_ expression via NIK, IKKα, and IKKβ by transfection with respective siRNAs. Transfection with NIK, IKKα, or IKKβ siRNAs knocked down their own protein levels and subsequently attenuated the TNF-α-induced cPLA_2_ expression (**Figure 5A**). To ascertain whether NF-κB participated in TNF-α-induced cPLA_2_ expression, a selective pharmacological inhibitor of NF-κB, Bay11-7082, was used for this purpose. Pretreatment with Bay11-7082 significantly reduced the TNF-α-induced cPLA_2_ protein, mRNA, and promoter activity (**Figures 5B,C**). Moreover, p65 siRNA was used to confirm the role of NF-κB in TNF-α-mediated effects. In addition, transfection with p65 siRNA also reduced TNF-α-induced cPLA_2_ expression (**Figure 5D**) and PGE_2_ secretion (**Figure 5E**) in these cells. The non-canonical NF-κB pathway is activated through particular TNF-α receptors that bind to the TRAF2 which leads to translocation of NF-κB into nucleus and induction of target gene expression (Tak and Firestein, 2001). Therefore, the translocation of NF-κB p65 was observed by an immunofluorescence microscope. Our findings showed that TNF-α time-dependently stimulated NF-κB p65 translocation from the cytosol into the nucleus fractions (**Figure 5F**) together with enhancing NF-κB promoter activity (**Figure 5G**). These results suggested that TNF-α-induced cPLA_2_ expression is mediated through NIK, IKKα/β, and NF-κB in these cells.

### TNF-α Induces NIK/IKKα/β-Dependent NF-κB Activation

**FIGURE 6:**
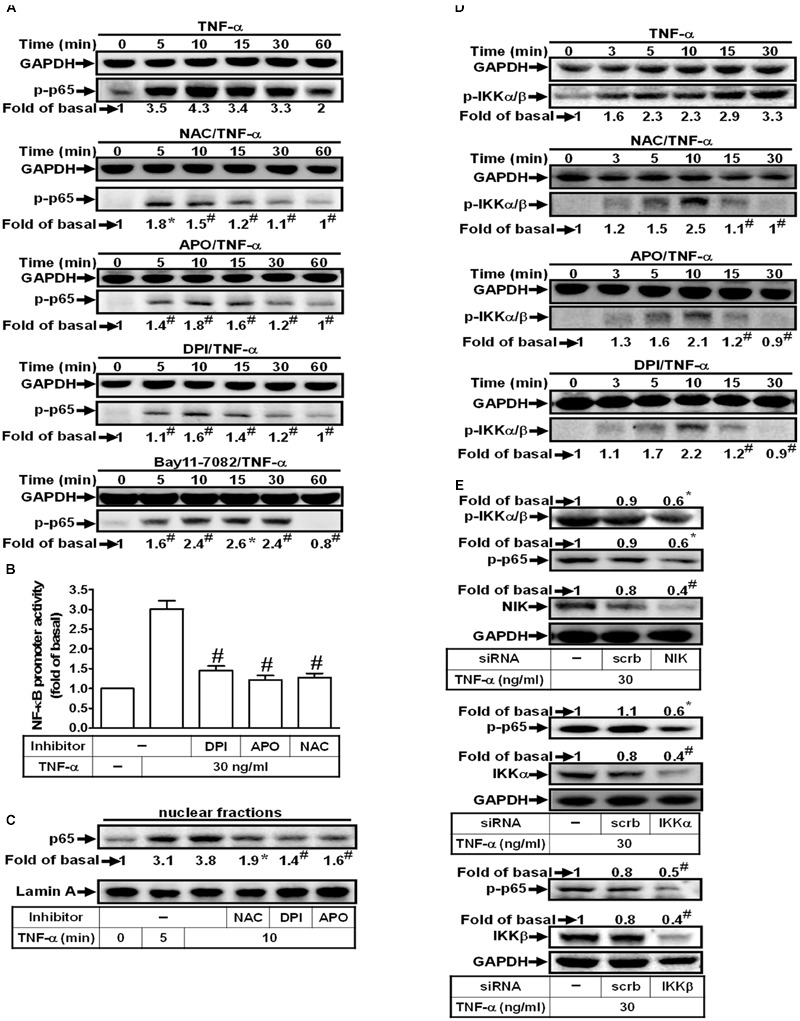
**TNF-α induces NIK/IKKα/β-dependent NF-κB activation. (A)** HPAEpiCs were pretreated with NAC (10 mM), DPI (1 μM), APO (100 μM) or Bay11-7082 (10 μM) for 1 h, and then treated with TNF-α for the indicated time intervals. The levels of p65 phosphorylation were determined by Western blot. The raw data of the GAPDH of **(A)** were provided with a supplementary data (Supplementary Figure S2). **(B)** Cells were pretreated with NAC, DPI, or APO for 1 h, and then treated with TNF-α for 2 h. NF-κB promoter activity was determined. **(C)** Cells were pretreated with NAC, DPI, or APO for 1 h, and then treated with TNF-α for the indicated time intervals. The nuclear fractions were prepared and analyzed by Western blot using an anti-p65 antibody. Lamin A was used as a marker protein for nuclear fractions. **(D)** Cells were pretreated with NAC, APO, or DPI for 1 h, and then treated with TNF-α for the indicated time intervals. The levels of IKKα/β phosphorylation were determined by Western blot. **(E)** Cells were transfected with scrambled, NIK, IKKα, or IKKβ siRNA, and then incubated with TNF-α for 10 min. The protein levels of phospho-IKKα/β, phospho-p65, NIK, IKKα, and IKKβ were determined. Data are expressed as mean ± SEM of three independent experiments. *^∗^P* < 0.05, ^#^*P* < 0.01 as compared with the cells exposed to TNF-α alone.

In this study, we investigated whether TNF-α stimulates NF-κB p65 activation via an NADPH oxidase/ROS-dependent pathway. As shown in **Figure 6A**, TNF-α markedly stimulated NF-κB p65 phosphorylation which was inhibited by NAC, DPI, APO or Bay11-7082. In addition, pretreatment with NAC, DPI, or APO also inhibited TNF-α-induced NF-κB promoter activity and NF-κB p65 translocation in HPAEpiCs (**Figures 6B,C**). On the other hand, we also observed that TNF-α significantly stimulated IKKα/β phosphorylation in a time-dependent manner, which was reduced by NAC, DPI or APO (**Figure 6D**). Finally, we investigated whether TNF-α stimulates NF-κB p65 phosphorylation via an NIK/IKKα/β pathway. As shown in **Figure 6E**, transfection with NIK siRNA attenuated the TNF-α-induced IKKα/β and NF-κB p65 activation. In addition, transfection with siRNA of IKKα or IKKβ markedly inhibited TNF-α-induced NF-κB p65 phosphorylation (**Figure 6E**). These results suggested that TNF-α stimulates NF-κB p65 activation through an NIK/IKKα/β-dependent cascade in HPAEpiCs.

## Discussion

**FIGURE 7:**
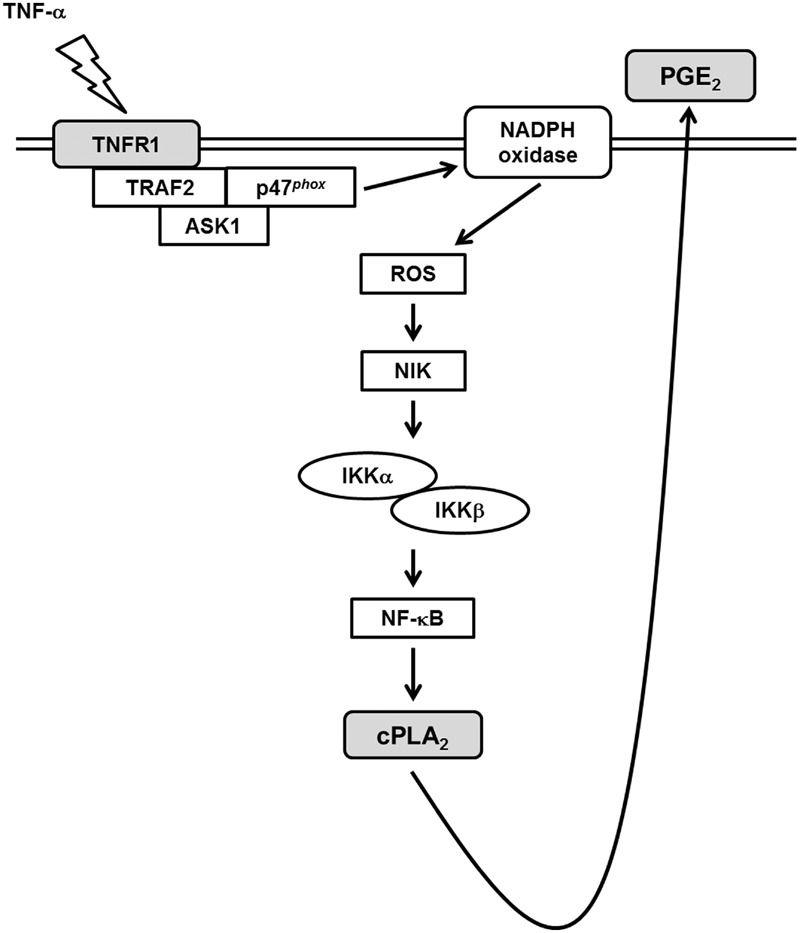
**Schematic representation of the signaling pathways involved in the TNF-α-induced cPLA_2_ expression in HPAEpiCs.** The mechanisms underlying TNF-α-mediated activation of TNFR1/TRAF2/ASK1/p47*^phox^*-dependent NADPH oxidase is required for the expression of cPLA_2_ in HPAEpiCs. Finally, the activation of ROS/NIK/IKKα/β/NF-κB pathway led to cPLA_2_ gene transcription and PGE_2_ release.

Various degrees of inflammation and tissue remodeling are characteristics of different pulmonary disorders including asthma and COPD. Expression of cPLA_2_ by mesenchymal cells in several extra-pulmonary sites contributes to the production of PGE_2_ which functions as biologically active lipid mediators in inflammatory responses (Khanapure et al., 2007). TNF-α has been shown to activate cPLA_2_ gene and involves in the late-phase airway hyperresponsiveness and inflammation (Choi et al., 2005), but few studies address the intracellular signaling pathways leading to its expression. It is showed that TNF-α activates ASK1, NADPH oxidase/ROS, IKKα/β, and NF-κB pathways in several cell types (Lee et al., 2009, 2011; Byeon et al., 2012). However, whether these signaling molecules participated in cPLA_2_ expression in TNF-α-treated HPAEpiCs was not completely defined. In this study, **Figure 7** addressed that pretreatment with the inhibitors of NADPH oxidase (APO and DPI), ROS (NAC), or NF-κB (Bay11-7082) or transfection with siRNA of ASK1, TRAF2, p47*^phox^*, NIK, IKKα, IKKβ, or p65 attenuated TNF-α-induced cPLA_2_ expression and PGE_2_ production in HPAEpiCs. Our studies confirmed that activation of NADPH oxidase, ROS, NIK, IKKα/β, and NF-κB via association of TNFR1, TRAF2, ASK1, and p47*^phox^* may be essential for cPLA_2_ expression in TNF-α-stimulated HPAEpiCs. cPLA_2_ induction and TNF-α-induced complex formation of TNFR1, TRAF2, ASK1, and p47phox were blocked by pretreatment with TNF-α neutralizing antibody. These results suggested that up-regulation of cPLA_2_ by TNF-α is, at least in part, mediated through the cooperation of TNFR1, TRAF2, ASK1, and NADPH oxidase leading to ROS generation and ultimately activates NF-κB pathway.

Several reports indicate that TNF-α may regulate inflammatory protein expression via activating various downstream protein kinases (Lin et al., 2004; Lee et al., 2009). Effects of TNF-α are achieved by binding to one of two distinct receptors, known as TNFR1 and TNFR2 (Lee et al., 2009). However, it is reported that TNF-α activates the proinflammatory or the programmed cell death pathways leading to tissue injury via binding to TNFR1 (van Vliet et al., 2005; Lee et al., 2009). In contrast, TNFR2 is involved in promoting tissue repair and angiogenesis (Bradley, 2008). Indeed, transfection with TNFR1 siRNA attenuated NADPH oxidase and ROS generation, revealing that TNFR1 plays a key role in modulating inflammatory responses in TNF-α-stimulated HPAEpiCs.

Reactive oxygen species are generated by various enzymatic reactions and chemical processes or can directly be inhaled from environment (Lee and Yang, 2012). Formation of ROS takes place constantly in every cell during normal metabolic processes. Activated phagocytic cells could produce large amounts of ROS induced by inhaled particles, microorganisms, or other mediators leading to the activation of the membrane-bound NADPH oxidase complex and the generation of superoxide anion (Lee and Yang, 2012). NADPH oxidase is recognized to be a key player in the generation of ROS when the cells or tissues exposed to various insults. Upon activation of NADPH oxidase by various stimuli, the complex of catalytic subunit (gp91^phox^) and p22^phox^ is anchored in plasma membrane which assembles with the regulatory subunits (p47^phox^, p40^phox^, p67^phox^, and small GTPase Rac) distributed in cytoplasm and leading to ROS generation (El-Benna et al., 2008). The initiation of the assembly NADPH oxidase is first dependent on the phosphorylation of p47^phox^ triggered by various activated protein kinases. Thus, p47^phox^ plays a role in the membrane translocation and ROS generation. Indeed, our results confirmed that blockage ROS accumulation either by a ROS scavenger (NAC), the inhibitors of NADPH oxidase (DPI and APO) or transfection with p47*^phox^* siRNA significantly attenuated TNF-α-induced cPLA_2_ expression and PGE_2_ synthesis. DPI or APO pretreatment reduced TNF-α-stimulated ROS generation. These results revealed that TNF-α increased cPLA_2_/PGE_2_ expression via NADPH oxidase-dependent ROS generation. p47*^phox^* is phosphorylated on several serine residues within the polybasic region of the protein, and these multiple phosphorylation events induce conformational changes that permit p47*^phox^* to interact with the cytoplasmic tail of p22*^phox^* and to initiate the formation of the active oxidase (Dang et al., 2006). In lung endothelial cells, Src regulates tyrosine phosphorylation of p47*^phox^* in hyperoxia-induced activation of NADPH oxidase and generation of ROS (Chowdhury et al., 2005). Moreover, in HPAEpiCs, we found that TNF-α could stimulate both serine and tyrosine phosphorylation of p47*^phox^*, and then promotes p47*^phox^* translocation from the cytosol to the membrane.

TRAF2 plays a central role in the cellular responses to stress and cytokines via their regulation of stress kinases, resulting in the activation of key transcription factors, including NF-κB, c-Jun, and ATF2. Upon exposure to TNF-α, TRAF2 is recruited directly to TNFR2 or via TRADD to TNFR1, which results in the activation of JNK1/2, p38 MAPK and NF-κB (Cabal-Hierro and Lazo, 2012). However, our previous report indicated that Jak2 also mediates the TNF-α-induced cPLA_2_ expression (Yang et al., 2014). To evaluate the relationship between Jak2 and MAPKs pathways, we have performed some more experiments to determine whether there is any connection between MAPKs and Jak2 using pharmacological inhibitors (AG490, SB202190, SP600125 and U0126). We found that pretreatment with AG490 had no effect on TNF-α-stimulated MAPKs phosphorylation, or with MAPK inhibitors (SB202190, SP600125 and U0126) failed to inhibit Jak2 phosphorylation (Supplementary Figure S1). These results suggested that Jak2 and MAPKs are independent pathways to mediate the TNF-α-induced cPLA_2_ expression. Here, we also found that TNF-α-induced cPLA_2_ expression and PGE_2_ release were markedly inhibited by blockage TRAF2. In addition, we also observed that TNF-α induced TNFR1 and TRAF2 complex formation. Previous study indicated that TNF-α stimulated the formation of a TNFR1/c-Src/p47*^phox^* complex in human airway smooth muscle cells (Lee et al., 2009). Here, we demonstrated that TNF-α induced TNFR1, TRAF2, and p47*^phox^* complex formation which was blocked by pretreatment with TNF-α neutralizing antibody, and leading to cPLA_2_ expression in HPAEpiCs.

ASK1, a member of the MAP3K family, regulates the activation of MAPK kinase 4 (MKK4)/MKK7-JNK and MKK3/6-p38 pathways. ASK1 can be activated by various types of stresses, such as ROS, TNF-α, and ER stress, and exerts pivotal roles in regulating cell apoptosis, differentiation, and inflammation (Fujino et al., 2007). Therefore, unregulated ASK1 activation is tightly related to various diseases. Moreover, we found that inhibition of ASK1 markedly reduced TNF-α-induced cPLA_2_ expression and PGE_2_ release. TNF-α also stimulated ASK1 phosphorylation in these cells. Upon exposure to ROS, Trx dissociated from the N-terminal of ASK1, which then became fully activated by recruitment of TRAF2 and TRAF6 (Fujino et al., 2007). Indeed, we found consistent results that TNF-α induces TRAF2 and ASK1 complex formation which is also blocked by pretreatment with TNF-α neutralizing antibody. In this study, we are the first to show a novel role of TNFR1/TRAF2/ASK1/p47*^phox^* complex formation in TNF-α-induced NADPH oxidase activation and ROS production in HPAEpiCs. In the future, we will further determine which domains of TNFR1, TRAF2, ASK1, and p47*^phox^* are involved in protein-protein interactions caused by TNF-α. Although ROS have been shown to regulate ASK1 activation (Fujino et al., 2007), in this study, we emphasized the critical role of ASK1 in TNF-α-induced ROS generation. Thus, in addition to the role of ROS in ASK1 activation, our results supported that the opposite hierarchical relationship exists between ROS and ASK1. Therefore, NADPH oxidase/ROS may act both as upstream regulators and downstream effectors of ASK1 in various cell types.

The NF-κB/Rel family complex, a redox-sensitive transcription factor, participates in controlling expression of many inflammatory genes (Sun, 2011). NF-κB usually forms herterodimer by p50 and p65/RelA subunits. In resting cells, nuclear translocation signal of NF-κB is masked by binding with an inhibitor protein called inhibitory κB (IκB) as an inactive non-DNA-binding form. Upon the stimulation by various NF-κB inducers, two serine residues of IκBα is phosphorylated, which then being ubiquinated by E3 ubiquitin-ligases (E3RSIκB) and subsequently degraded by the 26S proteasome (Sun, 2011). The released NF-κB dimers can then translocate into the nucleus and bind to the κB elements on the promoter of target genes. NIK was first identified as a TRAF2 interacting protein (Tak and Firestein, 2001). In inactive form, NF-κB transcription factors binds with the inhibitory proteins IκBs. IKKα and IKKβ regulates the phosphorylation of IκB proteins, which then being ubiquitination and degradation leading to nuclear localization of NF-κB transcription factors (Sun, 2011). In this study, inhibition of NIK, IKKα, IKKβ, and NF-κB markedly inhibited TNF-α-induced cPLA_2_ expression. Various extracellular stimuli such as TNF-α and IL-1β, viruses and environmental particulates (PM10s), and oxidative stress regulate the activation of NF-κB (Sun, 2011). Here, we reported that TNF-α time-dependently induced phosphorylation and translocation of NF-κB p65 and NF-κB promoter activity via an NADPH oxidase/ROS pathway. Otherwise, we also proved that TNF-α significantly induced IKKα/β phosphorylation via an NIK-dependent signaling, and then promoted NF-κB activation. Thus, we recognized that TNF-α-induced ROS generation may promote activation of the NIK/IKKα/β/NF-κB pathway in HPAEpiCs.

COX-2 and cPLA_2_ are tightly regulated by various mediators in several species (Beasley, 1999; Ali et al., 2008; Pavicevic et al., 2008). cPLA_2_ hydrolyzes the membrane phospholipids, resulting in the release of AA, which is further converted by the constitutive enzyme COX-1 or by the inducible COX-2, and PG synthases to biologically active PGs (DeWitt, 1999). On the other hand, several reports indicated that the levels of PGE_2_ are also degraded by an important enzyme, 15-hydroxyprostaglandin dehydrogenase (15-PGDH) to regulate the levels of PGEs (Pomini et al., 1999; Otani et al., 2006; Thiel et al., 2009). In our previous study, up-regulation of COX-2 in TSMCs has been shown to enhance PGE_2_ synthesis induced by LPS (Luo et al., 2003). Moreover, we also provided insights into the correlation between COX-2 and cPLA_2_ expression in ATPγS-stimulated VSMCs with similar molecular mechanisms and functional coupling to amplify the occurrence of vascular inflammation (Lin et al., 2009). PGE_2_ treatment also induced cPLA_2_ expression in VSMCs, which exerted as a positive feedback regulator in this response (Lin et al., 2009). Therefore, the synthesis of PGE_2_ could be a good index of AA release that is more sensitive than [^3^H]AA mobilization (Berenbaum et al., 2003) and PGE_2_ may further induce cPLA_2_ expression to amplify the occurrence of pulmonary inflammation. In this study, although the effect of PGE_2_ on COX-2 expression was not investigated, we tested the effect of TNF-α on PGE_2_ synthesis as a parameter of cPLA_2_ activity which may be an important issue for further study in HPAEpiCs.

## Conclusion

According to the literature reports and our results, **Figure 7** addresses a model for the molecular mechanisms of TNF-α-stimulated cPLA_2_ expression and PGE_2_ release in HPAEpiCs. To our knowledge, this is the first study to indicate that in HPAEpiCs, TNF-α-regulated activation of TNFR1/TRAF2/ASK1/p47*^phox^*-dependent NADPH oxidase is required for the expression of cPLA_2_. Finally, activation of the ROS/NIK/IKKα/β/NF-κB pathway leads to cPLA_2_ gene activation and expression. It is an important link for TNF-α-regulated cPLA_2_ expression in the pathogenesis of lung inflammatory diseases. Therefore, uncovering the signaling components in TNF-α-mediated cPLA_2_ expression in HPAEpiCs is important to develop new therapeutic strategies in pulmonary diseases.

## Author Contributions

C-CL, W-NL, R-LC, C-yW, L-DH, and C-MY substantially contributed to the conception or design of the work, the acquisition, analysis, and interpretation of data for the work. C-CL, W-NL, R-LC, C-yW, L-DH, and C-MY drafted the work and revised it critically for important intellectual content. C-CL, W-NL, R-LC, CyW, L-DH, and C-MY finally approved the version to be published. C-CL, W-NL, R-LC, C-yW, L-DH, and C-MY agreed to be accountable for all aspects of the work in ensuring that questions related to the accuracy or integrity of any part of the work are appropriately investigated and resolved.

## Conflict of Interest Statement

The authors declare that the research was conducted in the absence of any commercial or financial relationships that could be construed as a potential conflict of interest.

The reviewer HH and handling Editor declared their shared affiliation, and the handling Editor states that the process nevertheless met the standards of a fair and objective review.
