# Supplementary material for: TNF-α-Induced cPLA2 Expression via NADPH Oxidase/Reactive Oxygen Species-Dependent NF-κB Cascade on Human Pulmonary Alveolar Epithelial Cells
Source: Front Pharmacol. 2016 Nov 25;7:447. doi: 10.3389/fphar.2016.00447 (PMC5122718; doi:10.3389/fphar.2016.00447)
Supplement: Supplementary file 1 [file Data_Sheet_1.docx]

Supplementary Figure 1 Legend

**TNF-α induces phosphorylation of Jak2 and MAPKs.** (A) HPAEpiCs were pretreated without (control) or with (A) AG490 (10 μM), (B) SB202190 (10 μM), (C) SP600125 (10 μM) or (D) U0126 (10 μM) for 1 h, and then treated with 30 ng/ml TNF-α for the indicated time intervals. The levels of Jak2, JNK1/2, p38 MAPK, and p42/p44 MAPK were determined by Western blot using respective phosphorylated antibody. The levels of GAPDH was used as an internal control.

Supplementary Figure 2 Legend

**TNF-α induces NIK/IKKα/β-dependent NF-κB activation.** (A) HPAEpiCs were pretreated with NAC (10 mM), DPI (1 μM), APO (100 μM) or Bay11-7082 (10 μM) for 1 h, and then treated with TNF-α for the indicated time intervals. The levels of GAPDH were used as an internal control. This is the raw data of Figure 6A and the sources of Figure 6A are indicated with red windows, respectively.
