# Supplementary figures and images for: TNF-α-Induced cPLA2 Expression via NADPH Oxidase/Reactive Oxygen Species-Dependent NF-κB Cascade on Human Pulmonary Alveolar Epithelial Cells
Source: Front Pharmacol. 2016 Nov 25;7:447. doi: 10.3389/fphar.2016.00447 (PMC5122718; doi:10.3389/fphar.2016.00447)

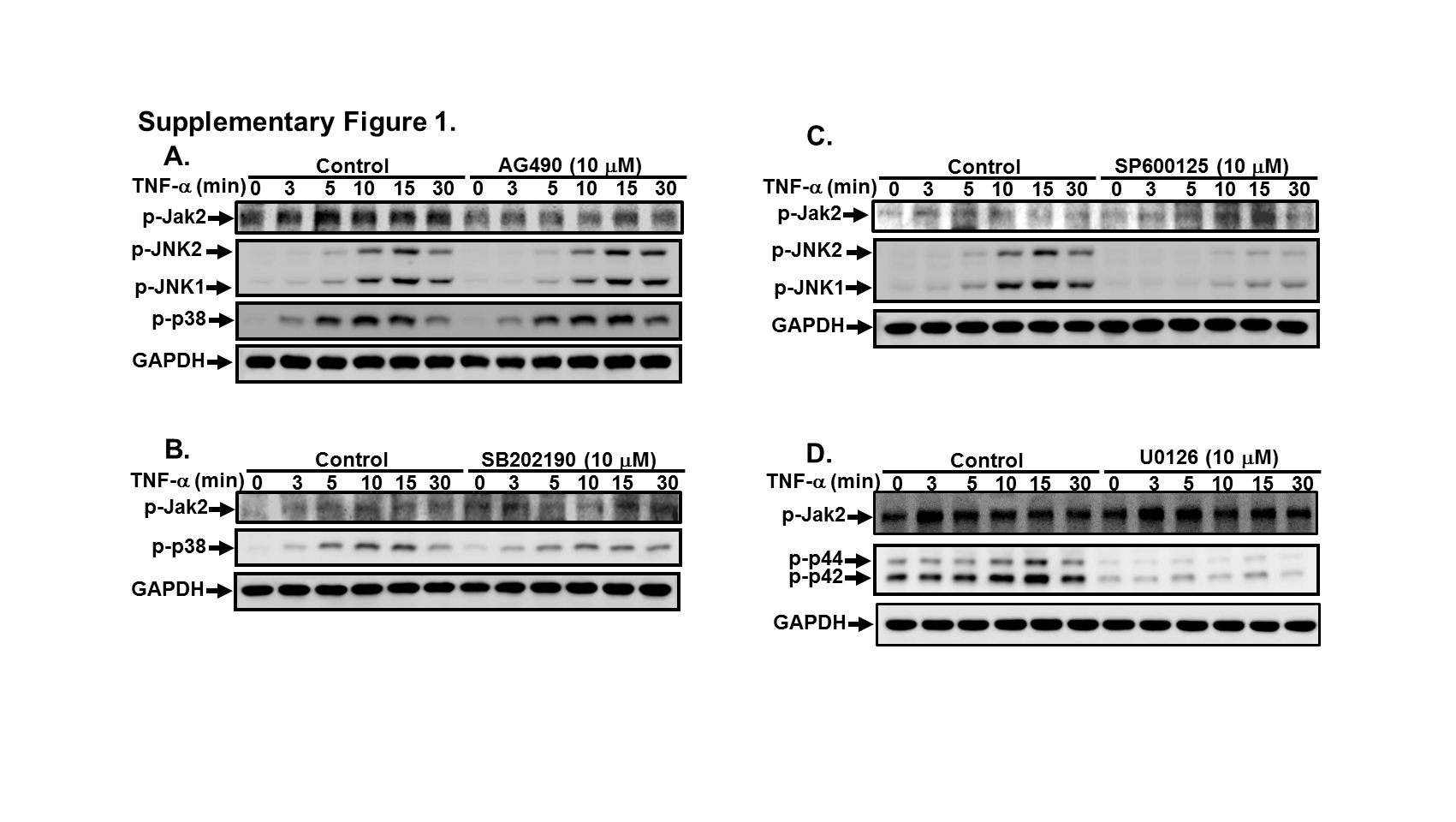

Supplement: Supplementary file 2 [file Image_1.TIF]

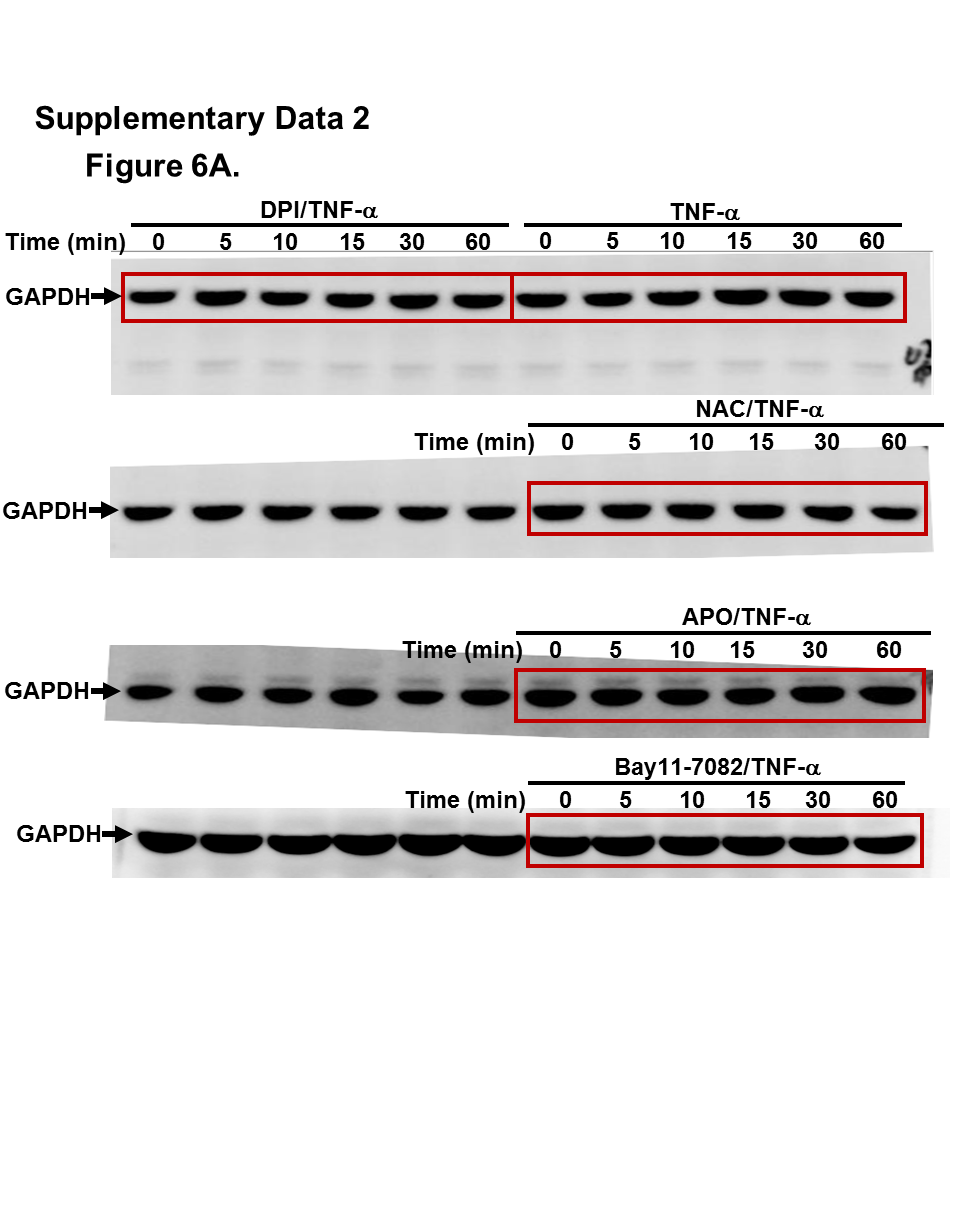

Supplement: Supplementary file 3 [file Image_2.TIF]
